# Operations-Vademecum für den praktischen Arzt

**Published:** 1900-03

**Authors:** 


**Operations-Vademecum für den praktischen Arzt.** Von Dr. Edmund Leser. Pp. viii. 190. Berlin: S. Karger. 1900.

In these pages there is a succinct account of modern surgical methods as practised by the author. General principles rather than minor details are discussed; but the book is profusely supplied with illustrations, which serve most usefully to exemplify and amplify the description of the surgical procedures.

